# Persistent Threats by Persistent Pollutants: Chemical Nature, Concerns and Future Policy Regarding PCBs—What Are We Heading For?

**DOI:** 10.3390/toxics6010001

**Published:** 2017-12-21

**Authors:** Bart Hens, Luc Hens

**Affiliations:** 1 College of Pharmacy, University of Michigan, 428 Church Street, Ann Arbor, MI 48109-1065, USA; barthens@umich.edu; 2 Department of Pharmaceutical and Pharmacological Sciences (KU Leuven), Herestraat 49, 3000 Leuven, Belgium; 3 Vlaamse Instelling voor Technologisch Onderzoek (VITO), Boeretang 200, 2400 Mol, Belgium

**Keywords:** polychlororinated biphenyls (PCBs), human exposure, biomonitoring, dioxins, accidents, PCB policy, regulations

## Abstract

Polychlorinated biphenyl (PCB)-contaminated sites around the world affect human health for many years, showing long latency periods of health effects. The impact of the different PCB congeners on human health should not be underestimated, as they are ubiquitous, stable molecules and reactive in biological tissues, leading to neurological, endocrine, genetic, and systemic adverse effects in the human body. Moreover, bioaccumulation of these compounds in fatty tissues of animals (e.g., fish and mammals) and in soils/sediments, results in chronic exposure to these substances. Efficient destruction methods are important to decontaminate polluted sites worldwide. This paper provides an in-depth overview of (i) the history and accidents with PCBs in the 20th century, (ii) the mechanisms that are responsible for the hazardous effects of PCBs, and (iii) the current policy regarding PCB control and decontamination. Contemporary impacts on human health of historical incidents are discussed next to an up to date overview of the health effects caused by PCBs and their mechanisms. Methods to decontaminate sites are reviewed. Steps which lead to a policy of banning the production and distribution of PCBs are overviewed in a context of preventing future accidents and harm to the environment and human health.

## 1. Introduction

Carcinogenic pollutants in the environment are of concern. Minimizing the risk they offer supposes reducing exposure. Recently, Hens et al. reviewed the dioxin incidents of the 20th century [1]. Their analysis showed the importance to identify sources of contamination and controlling them: it is essential that the lessons learned from the past will be converted in preventive measures addressing vulnerabilities in the future, while preventing new (major) contamination incidents. 

**Figure 1:**
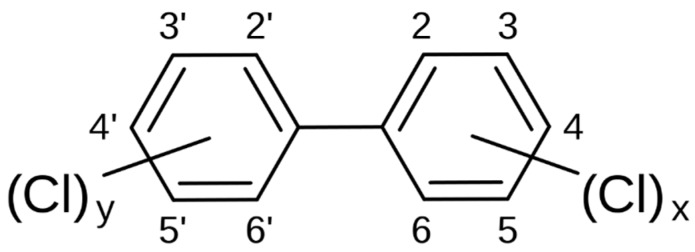
Molecular structure of polychlorinated biphenyls (PCBs) showing the 10 potential positions for chlorine to bind to.

Among the most important groups of pollutants with hazardous effects on human health are benzene-derivative structures, including polychlorinated benzenes, naphtalenes, biphenyls (PCB), polychlorinated dibenzo-*p*-dioxins (PCDD), and polychlorinated dibenzofurans (PCDF) [1,2,3,4]. The entire PCB family consists of 209 congeners. They vary in the number (1 to 10) and position of chlorine atoms on the biphenyl rings (Figure 1), and range in physical nature from oily liquids to waxy solids.

PCBs are stable molecules which are resistant to hydrolysis, oxidation, and temperature changes. They show excellent insulating properties, and are difficult to degrade, showing long half-life times in the environment. These characteristics are favorable for selected industrial applications (e.g., stabilizing additives in coatings and PVC plastics, paints, adhesives, and lubricants). However, mild oxidation of PCB leads to toxic PCDFs [5]. Physical and chemical properties of PCBs differ as the degree of chlorination increases: the more chlorine atoms attached to the molecule, the higher the lipophilicity and melting point, and the lower the solubility in water. Because of the lipophilic and hydrophobic properties, PCBs are highly soluble in most organic solvents, oils, and (animal) fat. The position of the chlorine atoms on the biphenyls, i.e., ortho (noncoplanar) versus non-ortho (coplanar) position, results in negligible or increased toxic effects, respectively, due to differences in the interaction with the aryl hydrocarbon receptor (AhR), which is an important regulator of biological responses in human organisms [6,7]. The interaction with AhR has been also observed for other dioxin-like compounds, as discussed before [1,8]. Briefly, the mode of action in human cells is, in most cases, regulated by disrupting/interfering the signal transduction pathway of a receptor, resulting in differentiation of tissue, cellular, and biochemical processes: after diffusion of 2,3,7,8-TCDD into the plasma membrane, it will bind to the AhR receptor. Subsequently, this activated complex binds to the transcription factor AhR nuclear translocator (Arnt) protein. Finally, the new complex “agonist–AhR–Arnt” enters the nucleus of the cell, and influences the expression of human genes by interfering with their promoter sequences.

From a pharmacokinetic point of view, these lipophilic compounds easily penetrate and bioaccumulate in fatty tissues, with intrinsic elimination half-lives ranging between months and years, depending on the chlorine substitution on the biphenyl groups [9,10]. The xenobiotic biotransformation of PCBs occurs by a phase I reaction, regulated by cytochrome P450, making the molecule more polar, which facilitates the excretion of the body. Depending on the position of the chlorine atoms (meta-para versus ortho-meta), the most appropriate cytochrome P450 enzyme (phenobarbital (PB)-induced P450s versus 3-methylcholanthrene (3MC)-induced P450s, respectively) catalyzes oxygenation and generates less lipophilic metabolites. Releases in the environment may occur from existing products, stockpiles, and waste and from industrial and non-industrial processes. Particular thermal processes also produce PCBs unintentionally; their signature clearly differs from a common PCB, produced by chlorinating biphenyl. In addition, previously released PCBs may be redistributed over time (e.g., from sediments and soils). They are persistent, and bioaccumulate in such way that they enter the food chain and accumulate in the fatty tissues of animals [1]. 

The major source of PCB exposure in humans is by intake of contaminated food. In industrialized countries in 1978, the daily intake of PCBs was estimated at 0.03 µg/kg/day, declining to less than <0.001 µg/kg/day by 1991 [9]. However, the consumption of fish from highly contaminated water bodies, such as the Great Lakes (the United States and Canada) significantly increases these figures [11]. PCB congeners in human organs, blood, and tissues can be measured using high-quality preparation and analysis, due to the evolution of highly sensitive analysis methods [12,13,14,15]. Measured concentrations in large-scale, longitudinal studies can be followed up and compared with regulated standards. Past incidents in Japan resulted in a stepwise, yet total ban on the production, use, and import of PCBs (1972). The domestic production of PCBs was banned in 1979 by the United States Congress, because of concerns on the toxicity and chemical stability of PCBs. This has led to establishing agreements and strategies aimed at controlling the pollution of, for instance, the Great Lakes: both Canada and the United States of America signed the first Great Lakes Water Quality Agreement that described how both countries avoid/reduce further pollution of the Great Lakes. In Europe, PCBs are listed as major persistent organic pollutants (POPs) and recognized to be harmful and toxic to human health. The Stockholm Convention list published in May 1995 indicated by that time, 12 POPs (among them, PCBs) that should be eliminated from the environment (http://www.pops.int). Throughout the years, this list was revised and updated [16].

This paper focusses on PCB incidents that occurred in the past decades, and discusses the impact of these incidents today: what are the levels of PCBs measured in humans/soils/sediments/open waters, and how are these levels related to the limits as indicated by regulatory agencies (e.g., World Health Organization (WHO))? Contemporary environmental legislation, in terms of guidelines and standards for PCB control and exposure, and measures to alleviate exposure, are reviewed [17], in combination with a discussion on the policy lessons learned from over 80 years of PCB pollution.

## 2. Materials and Methods: Literature Review

Papers published in peer-reviewed journals were searched in the PubMed database of the US National Library of Medicine National Institutes of Health, and selected on the keywords: polychlorinated biphenyls OR cancer OR America OR Midwestern states OR new york OR North Carolina OR South Carolina OR Great Lakes OR Dioxins OR PBC OR PCDF OR levels OR IARC OR Aroclor OR US EPA OR Washington OR Bloomington OR Massachusetts OR Europe OR New York OR incidents OR human biomonitoring OR PCBs. This resulted in 7,529,762 hits. As a first screen, only articles from 2000 onwards were considered. The next selection step was based on the apparent relevance of the title. After reading the abstract, a final selection was made. Articles published before 2000 were selected based on the snowball sampling technique. This resulted in a core number of 140 papers published in international, peer-reviewed journals, on which this review is based.

## 3. History and Accidents throughout the 20th Century

PCB was synthesized for the first time in 1881 in the laboratory, and has been produced industrially since 1929, first in the USA, and later, in many countries worldwide [18]. The total global production of PCB (excluding China and the USSR) between 1929 and 1988 is estimated at 1.5 million tons [19]. The United States of America was taking the lead with a production of 600,000 tons of PCB during the period 1930–1977 [9,20]. These numbers are not accurate, since some factories produced unregistered amounts of PCBs. As the major manufacturer of PCBs during the last century, the United States faced important incidents in different states during the period 1930–1977. Besides the United States, Europe had to face its own PCB problems as well.

Because of their chemical stability, PCBs were widely used as heat transfer liquids, transformer oils, and hydraulic fluids. One of the major producers during the period 1930 to 1977 in the United States, was the Monsanto Company (today, a multinational agrochemical and agricultural biotechnology company, Creve Coeur, MS, USA). This chemical company marketed PCBs as Aroclor^®^ [21]. Production of PCBs continued worldwide, and in particular, in eastern European countries until the mid-1980s. 

Already by 1899, chloracne, a painful disfiguring skin disease in PCB workers, was described [19]. Nevertheless, it took until 1979 before their toxicity and potential hazardous effects on human health were recognized by the US Congress and by the Stockholm Convention in 2001, after several industrial accidents. Different case studies of accidents, by that time, were linked to the exposure of PCBs. Categorizing PCBs as carcinogens by the International Association for Research on Cancer (IARC, 2016), was an important trigger for their wide recognition as hazardous products to human health, and their production ban [22].

More recently, an early warning that PCBs were widespread in the environment came from the work of Jensen et al. [23]. Working on DDT, they detected unspecified substances in the muscles of white-tailed sea eagles in Sweden. The levels were higher in these fish-eating birds than in the fish of the areas where they foraged. The conclusion being that these molecules were persistent in biological tissues, subject to bioaccumulation, and resistant to degradation. It took another two years before these substances were identified as PCBs.

Because of their pivotal role in the production of PCBs during the last century, many accidents occurred in different states around North America and the rest of the world; Table 1 lists 12 of the most known ones. 

The first incident mentioned in the table refers to PCB incidents at the Monsanto plant in Anniston, Alabama (1929–1971). PCB-containing materials leaked into Snow Creek. Afterwards, they spread to Choccolocco Creek, followed by a dump into the Logan Martin Lake. One study convincingly demonstrated that the increased levels of different congeners of PCB were positively correlated with increased serum levels of lipids in people living in Anniston, Alabama [24]. The increase in serum lipid levels (total cholesterol, LDL cholesterol, HDL cholesterol, triglycerides) is associated with an increased incidence of cardiovascular disease. Although accumulated PCB concentrations in fish declined since 2007, significant amounts of the PCBs accumulated in the sediments of the creeks and lakes. Following resuspension in the water, they re-enter the food chain. 

**Figure 2:**
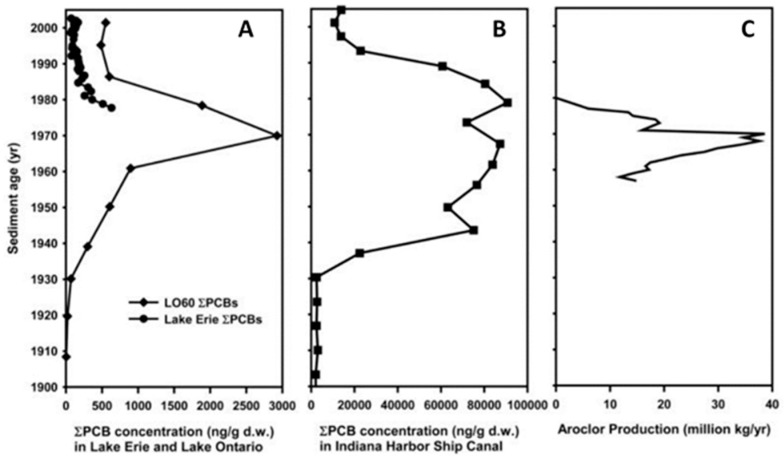
ΣPCB concentration trends in sediment from (**A**) Lake Erie and Lake Ontario, and (**B**) Indiana Harbor ship canal reflect production and use of commercial Aroclor mixtures in North America (**C**). Figure adopted from Hu and colleagues [21]. Copyright PMC 2011.

Although a lot of attention is given in the literature to the pollution by Aroclor^®^, other non-Aroclor^®^ PCB congeners were traced in the Great Lakes. They provide a record of long-term trends in environmental exposure. Figure 2 demonstrates the peak concentrations of Aroclor^®^ 1248 and 1254 in the seventies, as observed in Lake Erie, Lake Ontario, and in Indiana Harbor Ship Canal, which coincide with the production of Aroclor^®^ during those days [21].

**Figure 3:**
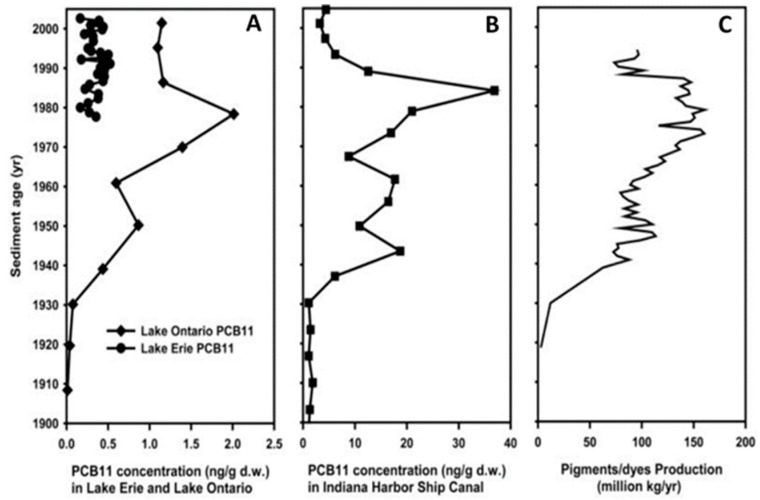
PCB11 concentration trends in sediment from (**A**) Lake Erie and Lake Ontario, and (**B**) Indiana Harbor ship canal reflect the production history of pigments/dyes production in the United States (**C**). Figure adopted from Hu and colleagues [21]. Copyright PMC 2011.

The non-Aroclor^®^ PCBs (PCB11, 206, 207, 208, and 209) reach a concentration peak in Lake Ontario and in the Indiana Harbor Ship Canal in the fifties, and a second peak in the seventies. These PCBs are associated with the production of commercial paint pigments and dyes, which were intensely used by that time (Figure 3). 

From the 1970s and 1980s, the concentrations of PCBs decline as a result of an intensive collaboration between state, provincial and local governments, and stakeholders from industry, academia, environmental and community groups, which led to a ban on the production and the use of PCBs, first in Sweden, and then the United States, and later on, in more than 60 countries which were participating the Stockholm Convention. In spite of declining concentrations, both the United States and Canada still discharge toxic residuals, such as PCBs and dioxins, in the Great Lakes [25].

Next to Monsanto, Westinghouse also produced substantial amounts of PCBs while manufacturing capacitors (1950–1977; Bloomington, Indiana). Bloomington is known for several PCB sources, and counts three US Environmental Protection Agency Superfund sites [26]. In 1993, PCBs were analyzed in air samples. These data were compared to measured concentrations during 1986–1987 at the same site. No statistical differences were observed during this 6 year period. During this period, an incinerator to decontaminate (1000 °C) PCB-polluted material was planned, but faced public opposition. However, in February 2008, Monroe County approved a plan to start cleaning up the three remaining contaminated sites by incineration and accumulation, at a cost of 9.6 million dollars.

Also, regions at the East Coast of the United States were contaminated by PCBs. Dumping of PCBs from 1947 until 1977 in Pittsfield (MA, USA) and Lockport (NY, USA) was largely the responsibility of General Electric [27,28]. Both areas are Superfund sites, listed to be decontaminated. One of the largest PCB dumps occurred in North Carolina in the summer of 1978. Over 31,000 gallons of PCB-contaminated oil were dumped along the roadsides of highways at night, the so-called “midnight dumps” [29]. The responsible organization behind these actions was the Ward PCB Transfer Company, which avoided, in this way, the increased expenses of chemical waste disposal, which were expected to be introduced on 2 August 1978. In South Carolina, the Sangama Weston plant (Pickens, SC, USA) was responsible for dumping PCB-contaminated wastewater into Twelve Mile Creek [30]. Two dams on the Twelve Mile Creek were dismantled, and the contaminated sediment was removed from the site.

The West Coast of the United States also deals with PCB-contaminated water bodies. In Washington state e.g., the Columbia River, the Duwamish River, Green Lake, Lake Washington, the Okanogan River, Puget Sound, the Spokane River contain high PCB concentrations, in particular, in the river soil. For the latter, it was shown that 44% originates from contaminated stormwater, and 20% from industrial discharges. Also, the Walla Walla River, the Wenatchee River, and the Yakima River are affected [31].

Not only the United States has to deal with PCB contamination of the environment. During the 1970s, impressive amounts of these chemicals were found entering the Netherlands from the Rhine River, which drains important industrial areas in Germany, France, and Switzerland. Surprisingly, the highest concentrations were, however, not found at the German–Dutch border, but in the sediments of the port of Rotterdam. Bioaccumulation in the food chain showed similar characteristics as in the Great Lakes [32].

In Münster (Germany), a screening program to control PCBs in breast milk showed their presence at the top of the human food chain. However, concentrations were rarely that high that breastfeeding should be given up [32]. Since 1987, the World Health Organization (WHO) coordinates an international study determining PCBs and dioxins/furans in the milk of lactating women (http://www.who.int/en/ and http://www.who.int/foodsafety/chem/POPtechnicalnote.pdf). Data from over 60 countries did not point to indications that breast milk feeding should be given up or limited—on the contrary. However, PCBs and PCDD/Fs are not a component that should be present in human milk. The data were merely used as indicators for the presence and the bioaccumulation of these persistent pollutants, and as a measure of the effectiveness of the phase-out policy for PCBs.

In France in 1974, the first food crisis occurred related to the contamination of soft cheese. The event provided an illustration of the differing views and priorities between the scientific and the regulatory agencies in the country [33]. As stated in the article of Narbonne and Robertson, it was clear that the “cheese” incident was a wake-up call for the French government: before the incident, the official position was that PCBs have only low toxicity to man, which was supported by several decades of experience in industry.

In January 1999, the chicken feed was contaminated with PCBs from a decommissioned electricity transformer in Belgium. The oil contained dioxins and furans. The problem was discovered after finding a declining hatching incidence in a chicken hatchery, and because of the emerging chick edema (swelling of tissue due to an increased fluid content). As originally no policy action was undertaken, the contamination spread over the whole food chain in Belgium and abroad, including popular items, such as mayonnaise and chocolates [3].

The occurrence of PCBs has been described in all environmental media (air, water, soil), (top) predators, and food in many industrialized and developing countries. In the latter, the importance of waste as a main source of PCBs is noticeable. In the Nigerian PCB inventory e.g., an estimated 3400 tons stem from waste, while PCB contaminated oil represents 421 tons, and the combined weight of PCB contaminated equipment accounts for 1061 tons [34].

Particular attention should be paid to places with high concentrations of PCBs (“hot spots”) as the water soil in ports, even in the Arctic [35].

Overall, even after the ban of PCBs, these data show the global, widespread, ubiquitous presence of PCBs in the environment, with the highest concentrations at the top of the human and the animal food chain.

## 4. Relationship between Exposure and Human Health: The Importance of Longitudinal Studies in Combination with *In vitro/In vivo* Research

Direct contact with PCBs results in atypical skin conditions, such as chloracne and rashes that were first observed among workers in PCB-producing companies, who previously were exposed to high concentrations of PCBs. Additional symptoms, such as a headache, ocular lesions, and irregularities of menstrual cycles were also demonstrated [22,36,37,38,39]. By the late 1930s, Monsanto was certainly aware of adverse health effects of occupational exposure to PCBs. In 1936, several of their workers were affected by chloracne. Three died, and the autopsies of two of them revealed serious liver damage. This latter finding was in line with the results of laboratory experiments with rats [40]. 

A historically well-known PCB incident with health impacts was a mass poisoning in Japan in 1968. Over 1800 people who ingested rice oil were affected. The oil contained large amounts of Kanechlor 400. The exposure resulted first in a skin disease with conjunctivitis, swollen eyelids, and chloracne all over the body. This was accompanied by pigmentation of nails, skin, and mucous membranes, increased sweating of the palms, severe headache, swollen joints, feelings of weakness, and in half of the victims, chronic bronchitis. Babies of mothers exposed during pregnancy were at birth “Coca-Cola^®^” (Atlanta, GA, USA) colored, growth retarded, and their nails were pigmented. The whole group of patients showed increased mortality due to malignancies of the liver, and the respiratory system [41,42,43].

Ten years after the Yusho outbreak, a similar problem of leakage of PCBs and furan/dioxin contaminated rice oil in the food chain caused large-scale human poisoning in Taiwan. Around 2000 people were intoxicated in 1978. Children born to mothers who were exposed during pregnancy were generally growth retarded, had dysplastic nails, antenatal teeth, spotty calcifications of the skull, wide open fontanels, and conjunctivitis. Many of them developed severe respiratory problems in their first year of life, and 20 percent died before the age of four [44]. Both the Yusho and the Taiwan groups showed decreased levels of serum IgA and IgM. A suppression of the cell-mediated immune system has been documented.

In general, exposure of pregnant women to PCBs showed mental and physical retardation in their offspring, as confirmed by animal studies in rats [40,45,46]. As mentioned in the introduction, the position of the chlorine atoms on the biphenyls, i.e., ortho (noncoplanar) versus non-ortho (coplanar) position, results in negligible or increased toxic effects, respectively, due to differences in interaction with AhR, as earlier discussed [6,7]. Quantitative structure–activity relationships (QSARs) within coplanar PCBs were determined by comparing their aryl hydrocarbon hydroxylase (AHH) induction potencies (EC50) in rat hepatoma H-4-II-E cells, and their binding affinities (ED50) for the 2,3,7,8-TCDD cytosolic receptor protein. Safe and colleagues clearly demonstrated that there was an excellent correlation between AHH induction potencies and receptor binding avidities of these compounds, and the order of activity was coplanar PCBs (3,3′,4,4′-tetra-, 3,3′,4,4′,5-penta-, and 3,3′,4,4′,5,5′-hexachlorobiphenyls) > 3,4,4′,5-tetrachlorobiphenyl ~ mono-ortho coplanar PCBs > di-ortho coplanar PCBs [47]. It was also apparent that the relative toxicities of this group of PCBs paralleled their biological potencies. The interaction with AhR has been also observed for other dioxin-like compounds, as discussed before [1]. Longitudinal studies, where serum PCB levels were followed up in individuals for several years, showed relationships between the systemic exposure to PCBs and neurological, immunological, lymphoreticular, genetic and endocrine effects, which are summarized in Table 2. This table provides an overview of different longitudinal studies that demonstrated the teratogenic/carcinogenic/mutagenic consequences of exposure to PCBs. All the information gathered by these studies helped the World Health Organization (WHO) to identify different levels of TEFs (Toxicity Equivalent Factors) for the different PCBs [17,48].

Listed as potentially carcinogenic by IARC (2016), exposure to congeners of PCB was directly associated with different types of cancer, such as cancer of the breast, prostate, and testicles. The potential for these molecules to interfere with DNA and cause damage was not only demonstrated by cross-sectional relationship studies, but also by *in vitro/in vivo* studies (animals) in the laboratory to confirm its hazardous effects on DNA (Table 3). The added value of *in vitro* studies that have been performed during the last decades helped competent international agencies to review and update the toxic equivalency factors (TEFs) as required. The TEF scheme and the toxic equivalents (TEQ) is based on endpoint effects (i.e., to an exposed individual), and because different congeners have quite different environmental fates, there is usually no simple relationship between the pattern of a source and the resulting exposure [1]. The idealized exposure in *in vitro* experiments might prove difficult to compare with the “real-life” exposure, taking into account the simultaneous presence of other congeners/carcinogenic molecules. In addition to the tested PCBs, other environmental carcinogens (e.g., dioxins) will contribute simultaneously to the same carcinogenic pathways. However, *in vitro* studies are indispensable to unravel the carcinogenic effects of toxic substances. *In vitro*–*in vivo* extrapolations should be carefully carried out, as the carcinogenic effect may be underestimated in real-life exposure, due to the presence of other toxic agents in the atmosphere. Currently, the list of TEFs is under revision, an action which should be undertaken every five years, updating the list with recent biochemical information [83]. 

## 5. Methods of PCB Destruction—Limiting Exposure

Destruction of PCBs is challenging, in view of their chemical stability. Incineration of PCBs at 1000 °C is the most common method to decontaminate soil and sediments. In theory, incineration will decompose PCBs, carbon dioxide, and hydrochloric acid. Another method is to add alkali metal carbonates, which will react and lead to pyrolysis [108]. Highly effective is thermal desorption for removing PCBs from soils [109]. Microbial degradation of soils is possible by adding *Shewanella oneidensis* to contaminated soils [110]. Also, other microorganisms, such as fungi (ligninolytic fungi), are described to degrade PCBs [111]. Although most of these techniques are still in process of research, they all have potential to destruct large amounts of PCBs in different soils/sediments/water bodies. Technology should focus on (i) cost-effectiveness, (ii) high selectivity, and (iii) time-saving methods to apply at contaminated sites worldwide. 

## 6. Regulations and Recommendations towards PCB Policy: Personal Perspectives

Establishing regulations limiting the exposure to PCBs was initially hampered by the negating attitude of main (industrial) stakeholders. In the late 1960s, when Monsanto was already, for decades, well aware of the occupational health effects of PCB exposure, the company, in its press communications, just denied the problem. They stated, e.g., that the claim made by Jensen et al. [23] about “highly toxic polychlorinated biphenyls” was simply not true. Negation of relevant facts also characterized later incidents, such as the 1999 feed and food PCB/dioxin poisoning in Belgium, where the competent authorities, four months after the initial contamination, decided for “acting at the lowest possible level”, administrative jargon for “doing nothing” [3].

By the early 1970s, Monsanto started an internal PCB policy. They reduced the amount of chlorine in the PCB mixtures, in particular, reducing the higher chlorinated PCBs. This was based on the idea that the higher chlorinated molecules were the most toxic—a concept which later proved too simplistic to alleviate the risks for the environment and human health. 

Shortly thereafter, governmental policies were also initiated. In 1972, responding to the reports on the presence and the effects of PCBs on the environment, the trophic chains, and food, Sweden banned these substances for “open uses” (sealants, paints, plastics) which resulted in direct releases in the environment. This marked the start of 45 years of PCB policy, which shows noticeable landmarks:

- After PCB use and manufacture were banned in 1979 (US), 1981 (UK), and 1986 (EU), PCB levels in biota started to decline (Jepson and Law, 2016), which shows the effectiveness of a ban policy.
- However, a complex and global problem of chemical pollution not only needs national policy actions, but also a worldwide response to internationally coordinated control measures. In February 1973, the Organization for Economic Co-operation and Development (OECD, 1973), was the first international organization advocating international action on polychlorinated biphenyls. Supported by the conclusions of the North Sea Ministerial Conferences, the OECD initiative cumulated in 2004, when the Stockholm Convention committed more than 90 signatory countries to phasing out or eliminating large stocks or other sources of the most hazardous persistent organic products (POPs), including PCBs.
- Since the 1970s, also, the knowledge base and the understanding of the mechanisms underlying the environmental health effects have deepened significantly. This particularly applies to the science supporting PCB policy design and decision making. The evolving list of health effects discussed in this paper provides an example. The first studies pointing to PCBs causing developmental effects, sexual maturation, and endocrine disruption date only from the 1980s. The elucidation of mechanisms that shed new light on the toxicology of the PCB congeners (e.g., PCB molecules with fewer chlorines are more susceptible to biotransformation) is even more recent.

The fundamental and applied PCB research during this almost half a century, also illustrates the progress in health risk assessment methods for mixtures. Originally PCB related risks were based on classical NOEL/LOEL data observed in experimental animals treated with commercial mixtures as Aroclors. In these experiments, mixtures were dealt with as a single compound. Later on, the health risk assessment became based on relative potency factors (TEF) which responded to the similar biological action of specific congeners. In this latter context, the activation of the aryl hydrocarbon receptor (AhR) has a pivotal role. We should not neglect human biomonitoring as an indicator and quantitative measure of exposure to environmental chemicals by measuring the chemicals or their metabolites in tissues or fluids (e.g., blood or urine) [112]. Moreover, in addition to human biomonitoring, a lot of attention goes out towards physiologically-based pharmacokinetic (PBPK) modeling, in order to predict tissue concentrations in humans to overcome ethical considerations [113]. Profiling concentrations of PCBs in biological samples have provided data to confirm environmental exposures and validate policy decisions. To date, multiple projects with respect to human biomonitoring are ongoing in different countries, to assist policy decisions regarding regulation.

The evolving approach has important practical consequences for a PCB policy and its interpretation. Guidelines and standards published before 2005 are largely outdated. The 2005 revision took into account much more information on fundamental action mechanisms and health endpoints than any other previous assessment [33]. 

In spite of the progress made in slowing down the increasing PCB contamination of the environment, these substances continue to threaten the survival of marine predators and human health. This shows that the current local, national, and international measures are insufficient. International concerted efforts remain needed, to limit the exposure. The experience of the past half century allows defining the contours of this upcoming PCB policy:

- Define indicators for both control and response variables that will contribute qualifying the exposure and monitor progress towards reducing the pollution. The WHO/United Nation Environment Program (UNEP) experience with monitoring PCBs in maternal milk is encouraging, but in view of the progressing knowledge on action mechanisms and health effects, likely incomplete, and too superficial and too simple to address this complex problem.
- Develop new technologies and social instruments that mitigate PCB pollution, emphasizing a preventive approach.
- Coordinate pollution control and sustainability efforts. Future policy should not only make use of the most recent significant scientific progress (which necessitates more flexibility than in the past) but should also opt for equilibrated long-term solutions.
- Facilitate multiple control efforts involving scientists, civil society, government, and international organizations.

These logical steps forward can only be realized in the context of continuing support by scientific research. In particular, the research in the realm of transferring and interpreting scientific data in a policy context should attract more attention. Too often, scientists and policymakers use different time scales, priorities, and endpoints to make a conversation and mutual understanding efficient. A practical and societally supported interpretation of the precautionary principle provides an example.

## 7. Conclusions

This paper shows a series of characteristics of PCBs in an environmental health context.

- By the 1930s, convincing occupational health evidence existed that PCBs could damage human health. Nevertheless, it took until the 1970s–1980s before a ban on these chemicals became gradually realized. With this almost half a century of latency, PCBs rank among the many chemicals for which early health warnings existed, while the policy was delayed. During this latency period, the (industrialized) world went through an impressive series of incidents, while an enormous environmental burden and delayed costs were built up. It is imperative for the future establishing mechanisms that this scientific evidence–policy latency period is shortened.
- As scientific data evolve and societal preparedness accepting policies increases, the PCB case shows that at least since the 1970s, a high level of proof existed pointing to the combined environmental and human health problems PCBs were responsible for. This environment–health nexus exists for many pollutants, and should be dealt with more intensively than before.
- PCBs illustrate the differentiated sources of pollution which necessitate a differentiated policy approach. While in OECD countries PCBs are closely associated with industrial pollution and a wide range of consumer applications, many developing countries face pollution from obsolete products and (illegal imported) waste. This necessitates not only international coordination, but also differentiation and flexibility of policies.
- The analysis of the health hazards and risks also entails elements of environmental health justice. Within one generation, the involuntary exposure of embryos and fetuses has different ethical consequences than the exposure of healthy industrial workers. The increasing insight into the genetic basis of PCB effects raises questions about how fair the next generations were treated during the past century.

## Figures and Tables

**Table 1:** Overview of major PCB contamination cases in a series of countries in the 20th century.

Year	State/Country	Source
1929–1971	Alabama, USA	PCB-containing materials were leached into Snow Creek, spread out over the Choccolocco Creek and Logan Martin Lake.
1929–1971	The Midwest States and Canada	Peak concentrations of Aroclor^®^ 1248 and 1254 in the seventies as observed in Lake Erie, Lake Ontario and in Indiana Harbor Ship Canal, similar to the production of Aroclor^®^ during those days.
1950–1977	Bloomington, Indiana	Westinghouse Electric Company produced large amounts of PCBs, which were still detectable in environmental sources (air and soils) until 1993.
1947–1977	Pittsfield, Massachusetts; Lockport, New York	Dumping of PCBs by General Electric, both sites now listed as Superfund sites to be decontaminated.
1978	North Carolina	One of the largest PCB dumps (31,000 gallons of PCB-contaminated oil) along the roadsides.
1970s	South Carolina	Sangama Weston Plant dumped PCB-contaminated waste into the Twelve Miles Creek.
1970s	Washington (State)	PCB contamination in river soil from industrial discharges.
1970s	The Netherlands	Contamination coming from industrial plants in Germany, France, and Switzerland into the Rhine River.
1970s–1980s	Germany	Immensely high concentrations of PCBs in the milk of breastfeeding women.
1974	France	Contamination of PCBs in soft cheese.
1999	Belgium	Chicken feed was contaminated with PCBs from a decommissioned electricity transformer.
1970s up until now	Nigeria	3400 tons of PCB-contaminated waste present in Nigeria throughout the years, produced by industrial processes.

**Table 2:** Summary of recent epidemiologic studies of human exposures to PCBs [9]. Copyright PMC 2016.

Health Effect/Outcome	Levels of Exposure	Outcome	Reference
Cancer/Non-Hodgkin’s lymphoma	PCBs and other organochlorines	PCBs 156,180, and 194 associated with increased risk of non-Hodgkin’s lymphoma	[49]
Cancer/Prostate cancer	30 PCBs and 18 organochlorine pesticide	PCB 180 was associated with an increase in risk of prostate cancer	[50]
Cancer/PSA levels	PCBs and other POPs (Chlordane, DDE)	In cases with PCB 153 > than the median concentration among controls, the OR = 3.15 (95% CI, 1.04–9.54)	[51]
Cancer/Prostate	30 PCB congeners in serum	Odds of high exposure group > twice that of lowest exposure group	[52]
Cancer/Prostate	Both high exposure to electromagnetic fields and PCBs	No association after adjusting for confounders	[53]
Cancer/Testicular/Seminoma	38 PCB congeners, DDT, hexachlorobenzene, chlordane	PCBs yielded odds ratio 3.8, 95% CI, 1.4–10 among case mothers	[54]
Cancer/Testicular Cancer	37 PCBs exposure	The concentrations of PCBs are higher in mothers to patients with testicular cancer	[55]
Developmental/Sensorineural hearing loss (SNHL)	2.8 μg/L serum total PCBs; mothers in 3rd trimester	The mean of mother’s serum PCB concentrations not related to the adjusted odds of SNHL	[56]
Development of natal and neonatal teeth	TEQ 11.9 pg/g fat PCDD/F, TEQ 7.24 pg/g fat	No association	[57]
Developmental; sexual maturation	PCBs (138, 153, 180), Dioxin-like compounds	Doubling of serum PCB 153 and dioxin-like chemicals significantly affected sexual maturation clarify	[58]
Developmental; reproduction effects	French cohort	At birth, cryptorchidism associated with higher prenatal exposure to PCBs.	[59]
Developmental/Age at menarche in offspring	PCBs and DDTs. Retrospective cohort study for two generations	No association with maternal PCB exposure	[60]
Developmental/Gingival health by standard dental indices and enamel by FDI index	Children living near industrial area contaminated with PCBs	Enamel defects in deciduous teeth significantly high in higher exposed children (Chi-Sq = 8.35; *p* = 0.03). For permanent teeth with any enamel defects (Chi-Sq = 7.237; *p* = 0.027). The extent of enamel defects is significantly greater in high PCB exposure group (Chi-Sq = 10.714; *p* = 0.005)	[61]
Developmental/Onset of menses	16 PCB congeners	PCBs levels are significant predictors of menarcheal status	[62]
Developmental/Visual function	Breastfed for 4 months and examined at 12 month of age	P100 with latency evoked potentials (VEPs) at 60 min. related to PCB 180 (*r* = −0.504)	[63]
Developmental/Dental enamel	Concentration of PBCs in diet	Enamel development defects were found in 71.3% exposed vs. 49.5% control	[64]
Developmental/Hormone levels and sexual differentiation	Prenatal exposure to PCBs. Umbilical cord specimens were collected.	20 boys with cryptorchidism; other 58 with spermaturia.	[65]
Endocrine/Type 2 diabetes mellitus	POPs/PCB153	Odds Ratio = 1.6; 95% CI, 1.0–2.7 associated with an increase of PCB-153 of 100 ng/g lipid	[66]
Endocrine/Type 2 diabetes mellitus	PCBs exposure	Positive linear association of PCB levels with diabetes at the time of enrollment in women	[67]
Endocrine/Thyroid	Retrospective study	Anti-GAD was 4 times higher than that of all controls	[68]
Endocrine/Testosterone and estradiol	PCBs concentrations 149 ng/g in blood and 177 ng/g in milk	Testosterone and estradiol levels were less in babies with high PCB concentrations	[69]
Endocrine/Thyroid and growth hormones	118 pregnant women (ages 25–34 years); Placental and cord blood samples. 12 dioxin-like PCBs	Significant negative associations between FT4, TSH and the increase of non-ortho PCBs (*r* = −0.2; *p* < 0.05)	[70]
Endocrine/Diabetes mellitus	PCBs 153	PCB 153 significantly associated with diabetes (an increase of 100 ng/g lipid corresponded to OR =1.16, 95% CI, 1.03–1.32, *p* = 0.03)	[71]
Endocrine/Diabetes mellitus/Insulin sensitivity	12 PCB congeners exposure	PCBs (123,126 and 169) were significant associated with insulin activity (*r* = −0.34, *p* < 0.05)	[72]
Endocrine/Type 2 diabetes	Persistent organic pollutants (POPs); 19 POPs in 5 subclasses	Association observed between HOMA-IR and two non-dioxin-like PCBs	[73]
Immunological/Antibodies to tetanus and diphtheria toxoids	Two cohorts from the Faroe Islands, mother serum (during pregnancy) and milk PCB levels were analyzed. Antibodies for tetanus and diphtheria were measured.	For each doubling of PCBs serum concentration, antibodies for diphtheria toxoid decreased by 24.4% at age 18 months (95% CI, 1.63–41.9; *p* = 0.04). Ab for tetanus toxoid decreased by 16.5% at age 7 y (95% CI, 1.51–29.3; *p* = 0.03)	[74]
Immunological/Rheumatoid arthritis	Cross-sectional study, 1721; 20 years or more of age; dioxin and non- dioxin-like (DL) PCBs	Odds ratios 1.0, 2.1, 3.5, and 2.9 across quartiles of DL PCBs. ODs for non-dioxin-like PCBs quartiles are 1.0, 1.6, 2.6, and 2.5. *p* for trends = 0.02. Men: no clear association	[73]
Immunological/Thymus atrophy	15 PCB congeners in neonates	Smaller thymus	[75]
Metabolism/Enzyme biomarker/	PCBs exposures via food (serum PCB concentrations)	Positive association with the serum levels of 9 PCB congeners	[76]
Musculoskeletal	This is part of the study of Swedish fisherman’s wives	No association found between PCB-153 and OH-PCBs and bone mineral density or biochemical markers of bone metabolism	[77]
Musculoskeletal/Bone mineral density (BMD)	Swedish fishermen and their wives	After adjustment for age and body mass index, the significant negative relationship between PCB-153 and BMD was not valid anymore	[78]
Musculoskeletal/Bone mineral density (BMD)	5 dioxin-like PCBs and 3 non-dioxin- like PCBs blood levels.	Male odds ratio negatively associated with BMD 1.6 (95% CI, 1.01–1.2) per 10 pg/mL CB-118	[79]
Musculoskeletal/Bone mineral density	Persistent organochlorines (PCBs, DDT)	PCBs were not associated with significant effects on bone density	[80]
Neurological/Neurodegenerative diseases.	PCB levels of workers were about 10 times higher than the PCB levels in community	Overall no significant effects (SMR = 1.40, 1.11, and 1.26, respectively. Women’s amyotrophic lateral (SMR = 2.26; 95% CI, 1.08–4.15)	[81]
Reproductive/Time to menopause	Halogenated biphenyl (PCBs, PBB) blood samples	No association with either PCBs or PBB	[82]

**Table 3:** Summary of health effects in animal studies on exposure to PCBs [9]. Copyright PMC 2016.

Species	Study Designs	Health Effects (Findings)	Reference
Rats (Sprague-Dawley)	Aroclor 1221 (0, 0.1, 1, or 10 mg/kg). In utero exposed female offspring (F1) and (F2); Gd 16 and 18	In both generations, litter sex ratio was skewed toward females	[84]
Mice (CD-1)	Aroclor 1016, fed 50 µg/kg/d; Gd 16–18; offspring examined at D3, D21, and D60	Increase prostate size, anogenital distance, decrease epididymal weight	[85]
Rats	Diets containing 0, 5, 20, or 40 mg PCBs/kg diet; exposure started 50 days before mating and terminated at birth	Reduced 1,25-dihydroxycholecalciferol during pregnancy	[86]
Rats (Sprague-Dawley)	2 years, Gavage, PCB 126. Control corn oil-acetone vehicle	Cytoplasmic vacuolation, chronic active inflammation, atrophy in exocrine pancreas	[87]
Rats (Sprague-Dawley)	125 ppm Aroclor 1254 in diet, pregnant rats	Reduce growth of hippocampal intra-and infrapyramidal (II-P) mossy fiber	[88]
Rats (22–24/dose) clarify	0 or 6 mg/kg A1254 (p.o. in corn oil) GD6-PND 21. Cross-fostered the offspring resulted in 4 groups (ctrl/ctrl; A1254/A/1254 perinatal exposure; A1254/ctrl, prenatal exposure only; ctrl/A1254, postnatal exposure only	Permanent hearing deficits in A1254/A1254 and ctrl/A1254 groups	[45]
Rats (Sprague-Dawley)	Females, gavage exposure to PCB 126	Bronchiolar metaplasia	[89]
Adult male rats	A1254 (diet) (30 mg/kg/day for 15 days)	Dehydrated PCB-fed rats had 863% increase in plasma vasopressin (VP); for the dehydrated control, a 241% increase in VP	[90]
Rats (Sprague-Dawley)	Single dose Gavage of 375 ug PCB 118/kg on GD 6	Hyperactivity and smaller testes, epididymides, seminal vesicles, decrease in sperm and spermatid numbers in offspring on PND 170.	[91]
Rats (females)	40 rats exposed to PCB 126 alone, vehicle, ovariectomy, or sham operation (2 × 2 factorial design) for 12 weeks	PCB 126 increases heart weight and serum cholesterol in both groups. PCB 126 increases blood pressure in sham-operated rats only.	[92]
Goat (kids)	Goat kids exposed to PCB 153 and PCB 126 during gestation and lactation. The average PCB concentrations in goat kids’ fat at age of 9 months were 5800 ng/g and 0.49 ng/g fat weight for PCB 153 and PCB 126 respectively.	At puberty, low LH, delayed puberty, higher progesterone level in the group exposed to PCB 153. PCB 126 has no effect at these levels	[93]
Pregnant Rats	0, 0.5, and 5.0 mg/kg bt of 4-OH-CB107 or Aroclor 1254 (25 mg/kg bt) during GD 10-GD16	At 0.5 and 5.0 mg of 4-OH-PCB 107 a significant prolongation of the estrous. A 50% increase in plasma estradiol levels in female offspring in animals treated with 5 mg 4-OH-CB107/kg body weight. Aroclor 1254 treatment had no significant effects on estradiol levels.	[94]
Female Rats (Sprague-Dawley)	Binary mixture of 1000 ng/kg PCB 126 + 1000 ng/kg PCB 153; PCB 126 = PCB 118 (216 and 360 ng TCDD equivalent/kg)	Hyperplasia of respiratory epithelium and metaplasia of olfactory epithelium, acute inflammatory exudates observed within the lumen of nasal cavity on the affected area	[87]
Long-Evans 5 day Pregnant Rats	2 or 4 mg/kg/subcutaneous injection of PCB 77 on GD 6–18	Nursing time was reduced in both treatments. At 4 mg/kg body weight, the amount of licking time and pup mortality were increased	[95]
Rats (Sprague-Dawley)	PCB 126 + PCB 153; PCB 126 + PCB 118; PCB 126 alone; PCB 153 alone; TCDD + PCB 126 + PeCDF; By Gavage/2 years	In all mixtures, the incidences of gingival squamous cell hyperplasia were increased significantly. In TCCD, PCB 126, and PCB 126 + PCB 153 treated groups, squamous cell carcinoma increased significantly.	[96]
Pregnant Rat	2 mg/kg PCB 77 Gd 6–18 and gestation	Increase frequency of nursing bouts and amount of maternal auto-grooming	[97]
Rats	PCBs on maternal odor conditioning in rat pups 12–14 days of age	Significantly depressed the preference for the maternal-associated cue, but did not impair discrimination for a novel odor	[46]
Rats	Pregnant rats administered single doses of PCB 132 at 1 or 10 mg/kg on a gestational day 15. Male offspring were assessed on postnatal day 84	Decreased cauda epididymal weight, epididymal sperm count, and motile epididymal sperm count in adult offspring. The spermatozoa of PCB 132-exposed offspring produced significantly higher levels of ROS than the controls. Low-dose PCB 132 group, p53 was significantly induced and caspase-3 was inhibited. High-dose group, activation of caspase-3 and -9 was significantly increased, while the expressions of Fas, Bax, bcl-2, and p53 genes were significantly decreased.	[98]
Rats	Noncoplanar PCBs were fed to rat dams during gestation and throughout three subsequent nursing weeks	Abnormal development of the primary auditory cortex (A1)	[99]
Female Goat	Goat dams were orally dosed with PCB 153 in corn oil (98 µg/kg body wt/day) or PCB 126 (49 ng/kg body wt/day) from day 60 of gestation until delivery. The offspring were exposed to PCB in utero and through maternal milk. The suckling period lasted for 6 weeks.	Perinatal exposure to PCB 153, but not PCB 126, resulted in altered bone composition in female goat offspring	[100]
Female Rats (Sprague-Dawley)	Daily oral administration of vehicle (corn oil) or 1 or 3 μg/kg of PCB-126 from 2 weeks prior to mating with intact males until 20 days after delivery, examined from birth until puberty	Direct effect on the ovary and adverse effects female puberty by altering the morphological and functional development of the female reproductive system	[101]
Adult Female Rats (Sprague-Dawley)	Prenatal exposure to the PCB mixture Aroclor 1221 on adult female	Mating trial pacing, vocalizations, ambulation, and the female’s likelihood to mate. Were these impaired?	[102]
Female Mice (C57BL/6)	Temporal analysis, mice were orally gavaged with PCB126 or sesame oil as the vehicle and sacrificed after 2, 4, 8, 12, 18, 24, 72, 120, or 168 h. In the dose-response study, mice were gavaged with 0.3, 1, 3, 10, 30, 100, 300, 1000 μg/kg PCB126, 30 or 100 μg/kg TCDD and sacrificed after 72 h	251 and 367 genes were differentially expressed by PCB 126 at one or more time points or doses, respectively, significantly less than elicited by TCDD. At 300 μg/kg PCB 126 elicited a subset of weaker effects compared with 30 μg/kg TCDD in immature, ovariectomized C57BL/6 mice.	[103]
Rats	Pregnant rats were treated orally with PCB 126 at a dose of 30 µg/kg or corn oil, its vehicle, on gestational day 15, and their male offspring were subjected to locomotor activity and anxiety-related test, social interaction, and rotating test at 4–5 weeks old	% time spent in the center, social interaction time, and the number of rearing were significantly reduced in PCB treated group	[104]
Female Rats	Pre- and/or postnatal exposure to PCB 77. Pregnant rats were treated with oil or PCB dissolved in oil (2 mg/kg b.w.) on gestation days 6–18 and then given pups that had been exposed to either the oil vehicle or PCB during gestation. Female offspring were monitored until adulthood.	None of the treatments (preferred to exposed) affected female sexual behavior	[105]
Adult Rats	PCB mixture Aroclor 1254 (A1254) at 0.1 or 1 mg/kg/day in the maternal diet throughout gestation and lactation. Focal cerebral ischemia was induced at 6–8 weeks of age via middle cerebral artery occlusion, and infarct size was measured in the cerebral cortex and striatum at 22 hr of reperfusion.	Significantly decreased striatal infarct in females and males at 0.1 and 1 mg/kg/day, respectively. Effects of developmental A1254 exposure on Bcl2 and Cyp2C11 expression did not correlate with effects on infarct volume.	[106]
Mice	Single gavage dose (150 µmol/kg body weight) of PCB 77, PCB 104, PCB 153 (as a mixture)	Induction of pro-inflammatory mediators in livers, lungs, and brains. The strongest expression of pro-inflammatory proteins occurred 24 h following the PCB administration independent of the class of PCB.	[107]
